# Supplementary material for: Long-Read Sequencing Reveals Cell- and State-Specific Alternative Splicing in 293T and A549 Cell Transcriptomes
Source: Int J Mol Sci. 2026 Jan 3;27(1):487. doi: 10.3390/ijms27010487 (PMC12787195; doi:10.3390/ijms27010487)
Supplement: Supplementary file 1 [file ijms-27-00487-s001.zip › ijms-3990471-supplementary.pdf]

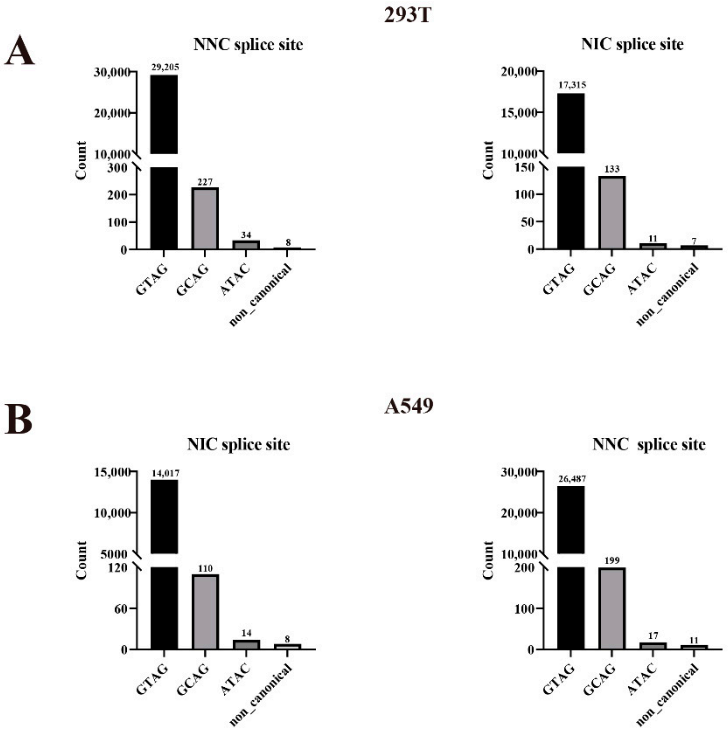

Supplementary Figure S1. The number of splice sites in 293T and A549 cell lines. GT-AT, GC-AG, and AT-AC are considered canonical types; all remaining types are consequently non-canonical.

A.

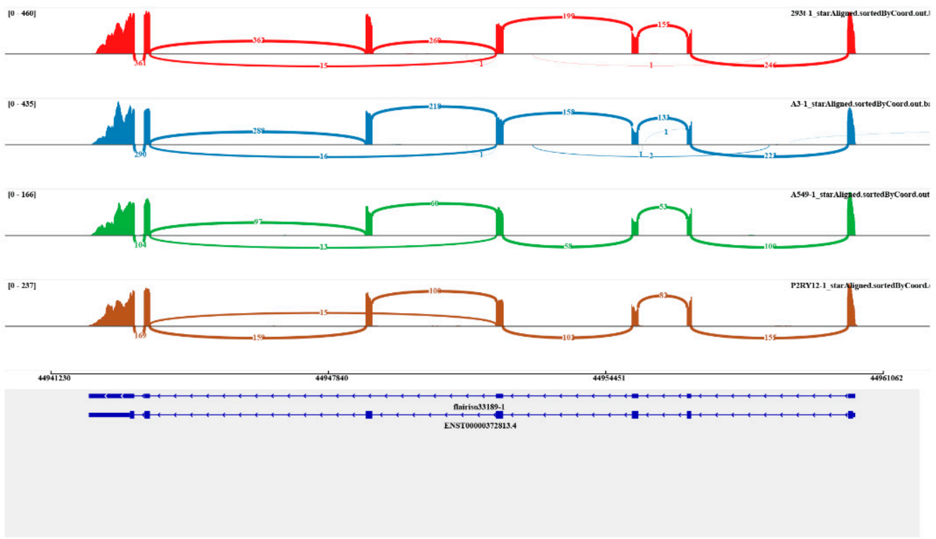

B.

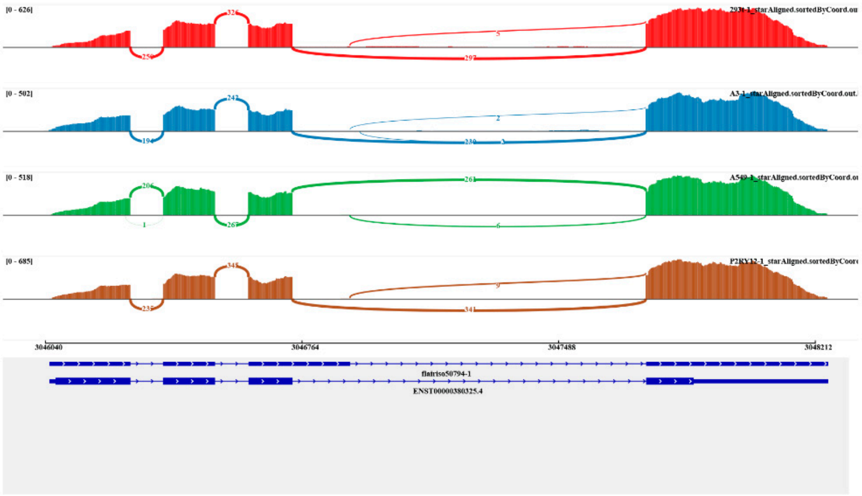

C.

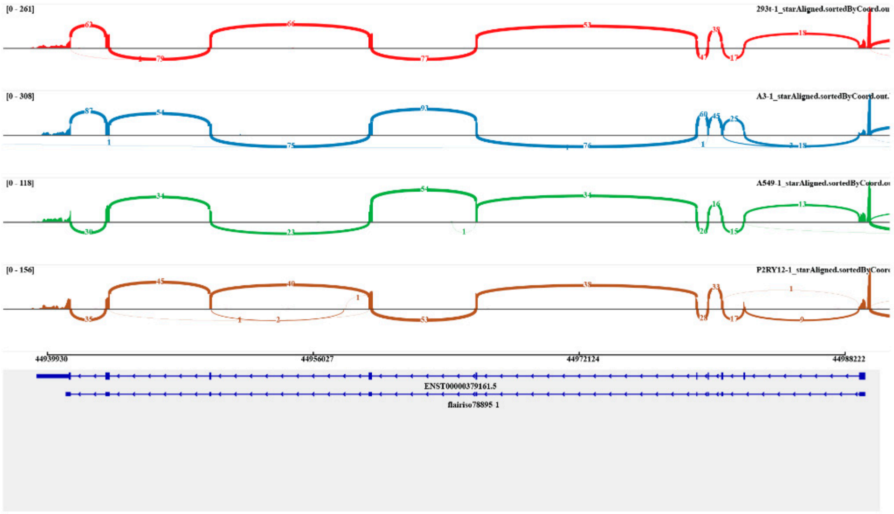

Supplementary Figure S2 (A) Sashimi plot of ENST0000372813 and its isoform flairiso33189-1. (B) Sashimi plot of ENST0000380325 and its isoform flairiso50794-1. (C) Sashimi plot of ENST0000379161 and its isoform flairiso78895-1.
